# Clinical Significance of BTLA, CD27, CD70, CD28 and CD80 as Diagnostic and Prognostic Markers in Ovarian Cancer

**DOI:** 10.3390/diagnostics12020251

**Published:** 2022-01-20

**Authors:** Janina Świderska, Mateusz Kozłowski, Maria Gaur, Ewa Pius-Sadowska, Sebastian Kwiatkowski, Bogusław Machaliński, Aneta Cymbaluk-Płoska

**Affiliations:** 1 Department of Gynecological Surgery and Gynecological Oncology of Adults and Adolescents, Pomeranian Medical University in Szczecin, al. Powstańców Wielkopolskich 72, 70-111 Szczecin, Poland; jasia.swiderska@gmail.com (J.Ś.); mtyskmr@yahoo.co.uk (M.G.); aneta.cymbaluk@gmail.com (A.C.-P.); 2 Department of General Pathology, Pomeranian Medical University in Szczecin, al. Powstańców Wielkopolskich 72, 70-111 Szczecin, Poland; ewapius@wp.pl (E.P.-S.); machalin@pum.edu.pl (B.M.); 3 Department of Obstetrics and Gynecology, Pomeranian Medical University in Szczecin, al. Powstańców Wielkopolskich 72, 70-111 Szczecin, Poland; kwiatkowskiseba@gmail.com

**Keywords:** BTLA, CD27, CD70, CD28, CD80, immune proteins, diagnostic biomarker, prognostic biomarker, ovarian cancer

## Abstract

It is very important to find new diagnostic and prognostic biomarkers. A total of 79 patients were enrolled in the study. The study group consisted of 37 patients with epithelial ovarian cancer, and the control group consisted of 42 patients with benign ovarian lesions. Five proteins involved in the immune response were studied: BTLA, CD27, CD70, CD28, CD80. The study material was serum and peritoneal fluid. The ROC curve was plotted, and the area under the curve was calculated to characterize the sensitivity and specificity of the studied parameters. Univariate and multivariate analyses were performed simultaneously using the Cox regression model. The cut-off level of CD27 was 120.6 pg/mL, with the sensitivity and specificity of 66 and 84% (*p* = 0.014). Unfavorable prognostic factors determined in serum were: CD27 (for PFS: HR 1.26, 95% CI 1.21–1.29, *p* = 0.047; for OS: HR 1.20, 95% CI 1.15–1.22, *p* = 0.014). Unfavorable prognostic factors determined in peritoneal fluid were: BTLA (for OS: HR 1.26, 95% CI 1.25–1.31, *p* = 0.033). We conclude that CD27 should be considered as a potential biomarker in the diagnosis of ovarian cancer. BTLA and CD27 are unfavorable prognostic factors for ovarian cancer.

## 1. Introduction

Epithelial ovarian cancer (EOC) remains a cancer with the highest mortality rate among gynecologic cancers. EOC is characterized by high specificity of disease spread through continuity in the pelvis and abdominal cavity. In 70% of patients presenting to the hospital, the tumor is in stage III with peritoneal dissemination of small tumors and varying degrees of ascites. Current reports indicate that even in advanced ovarian cancer, primary surgery without residual disease in the form of peritoneal carcinomatosis is most important. Despite total debulking surgery (up to R0) and subsequent platinum-based chemotherapy with taxanes, the vast majority of patients have relapses, often with intraperitoneal spread and ascites. The treatment of recurrent ovarian cancer is currently a challenge because once the cancer becomes chemo-resistant, there are few therapeutic options that can significantly affect overall survival. Therefore, new diagnostic and prognostic biomarkers, as well as new immune checkpoints of the body, continue to be sought to offer new solutions for patients with ovarian cancer. One such innovative concept, since the emergence of new technologies, seems to be immunotherapy in cancer [1]. It is known that a healthy human body also produces tumor cells, but these are continuously eliminated by the immune system [2,3]. The hypothesis of tumor immune surveillance has been put forward [4]. The main roles are played here by both T and B lymphocytes, which are responsible for recognition of foreign antigens and initiation of the organism response. The importance of coordinating antitumor response, combining the action of cytolytic T cells and antibody-producing cells in ovarian cancer, is indicated in studies by Kroeger et al. [5]. B and T lymphocyte attenuator (BTLA) is a co-signal protein with expression on T lymphocytes, B lymphocytes, natural killer (NK) cells and antigen-presenting cells [6,7]. BTLA belongs to the CD28 superfamily, which is a subgroup of immunoglobulins. Two members of this group are positive regulators of T cell function, namely CD28 and ICOS. In contrast, two others, CTLA-4 and PD-1, act as inhibitors of T lymphocyte function. The role of the BTLA protein is not fully understood, but it is thought to exert bidirectional regulatory effects: one identical to CTLA-4 and PD-1, as a T cell inhibitor [6], and the other stimulating T cells. There are reports that depending on the tumor microenvironment, the action of BTLA varies, hence its different expression depending on the tumor type. In more cases, it assumes T cell inhibitory activity. In addition, we have two signaling pathways that appear to be crucial to the body’s immune responses. These are the CD70/CD27 and CD80/CD28 pathways [8,9,10,11]. The receptor molecules CD27 and CD28 are found on T lymphocytes and the costimulatory molecules CD70 and CD80 are found on antigen presenting cells. These molecules are responsible for regulating immunity without producing tolerance, which can be produced by presenting antigen without an induced state. It should be noted that signaling through the CD27/CD28 receptor appears to be crucial for T cell expansion and survival [8]. To the best of our knowledge, immunological factors belonging to these groups have not yet been evaluated for their usefulness in diagnostic tests or as prognostic factors in patients with ovarian cancer.

The aim of the study was to evaluate the concentrations of BTLA, CD27, CD70, CD28 and CD80 in serum and peritoneal fluid in the study groups. The concentrations were also evaluated in selected subgroups of patients with ovarian cancer. The levels of serum and peritoneal fluid concentrations of the studied proteins were evaluated as potential relationships and correlations between the main groups studied. Finally, the studied proteins were evaluated as diagnostic and prognostic biomarkers of ovarian cancer.

## 2. Materials and Methods

### 2.1. Participants

#### 2.1.1. Participation in the Study

Initially, 107 patients aged 34–76 years presenting to the Department of Gynecological Surgery and Gynecological Oncology of Adults and Adolescents with ovarian cysts or tumors accompanied by peritoneal fluid were included in the study. Patients with acute pelvic inflammatory conditions, cirrhosis, circulatory insufficiency, renal insufficiency and autoimmune diseases were excluded from the study because of the possible influence of these conditions on the results of the proteins tested. In the final stage, 79 patients were included in the study. Each patient was thoroughly informed about the study. The women signed an informed patient consent to participate in the study. Depending on the ovarian pathology, the patients were divided into two groups. Group A included 37 patients with epithelial ovarian cancer and group B included 42 patients with benign ovarian lesions. The presence of ovarian pathology was confirmed by imaging studies and histopathological examination. 

#### 2.1.2. Characteristics of the Study Group

The median age of patients with ovarian cancer was 60.2 years, and among patients with benign ovarian lesions it was 47.6 years (*p* = 0.046). The median BMI of patients with ovarian cancer was 27.8 kg/m^2^, and among patients with benign ovarian lesions it was 25.2 kg/m^2^ (*p* = 0.081). In the ovarian cancer group, 11 (29.7%) patients were premenopausal, while 26 (71.3%) were postmenopausal. In the group of patients with benign ovarian lesions, 20 (47.6%) patients were premenopausal, while 22 (52.4%) were postmenopausal (*p* = 0.005). Patients with ovarian cancer were divided according to the histological subtype of the cancer, and serous carcinoma was the most common subtype (31 patients). Furthermore, patients with ovarian cancer were divided according to FIGO staging: 7 patients were at stage I–II, and 30 patients were at stage III–IV. Regarding the histological malignancy of the cancer (grading), 13 patients had low-grade cancer, and 24 patients had high-grade cancer. Patients with ovarian cancer were also divided according to the presence of neoplastic cells in peritoneal fluid, peritoneal carcinomatosis, type of debulking surgery (total and subtotal) and type of chemotherapy (neoadjuvant and adjuvant). The detailed characteristics of patients with epithelial ovarian cancer and benign ovarian lesions are shown in Table 1 and Table 2.

### 2.2. Instruments

#### 2.2.1. Pre-Laboratory Sample Preparation

During routine preoperative examinations, an additional 5 mL of whole blood was collected, which was immediately centrifuged and frozen to −70 °C. For primary surgery, blood was collected before surgery, whereas for neoadjuvant chemotherapy, blood was collected before the administration of chemotherapy. In addition, peritoneal fluid (approximately 6 mL) was collected during surgery and separated into two tubes. In patients who received neoadjuvant chemotherapy, peritoneal fluid was collected during the initial diagnostic laparoscopy. In the study groups, peritoneal fluid (in the form of ascites or fluid in the pouch of Douglas) was present in most patients. Only 4 patients with benign cystic lesions had no peritoneal fluid. In those patients who did not have peritoneal fluid, washing was performed, and the fluid was collected to assess the presence of tumor cells and to determine the concentrations of the proteins studied. This fluid was then processed by centrifugation at room temperature for 10 min (1000× *g* rotation) to remove impurities. The resulting material was frozen to −70 °C in two independent tubes. The material was then subjected to laboratory analyses.

#### 2.2.2. Laboratory Analysis—Multiplex Immunoassay

BTLA, CD27, CD70, CD28 and CD80 concentrations were quantified in serum and peritoneal fluid in groups of patients with epithelial ovarian cancer and benign ovarian lesions by multiplex fluorescent bead-based immunoassays (Luminex Corporation, Austin, TX, USA) using commercial Human Immuno-Oncology Checkpoint Protein Magnetic Bead Panel 1 (Merck Millipore, Billerica, MA, USA). An amount of 25 µL of each standard, control and samples, was added to the plate together with multiplex antibody capture bead solution, and the plate was incubated with shaking at 4 °C overnight. Next day, each well was washed with 200 µL Wash Buffer 3 times by using hand-held magnet. An amount of 25 µL of detection antibody cocktail was pipetted to each well, and the plate was sealed and incubated at room temperature for 1 h on a shaker. After this step, 25 µL streptavidin–phycoerythrin mixture was added to the plate and incubated with agitation for 30 min in the dark. Finally, after washing, the microspheres in each well were resuspended in 150 µL Sheath Fluid and shaken at room temperature for 5 min. The plate was then read and analyzed on the Luminex analyzer, and analyte concentrations were determined from five different standard curves showing MFI (Median Fluorescence Intensity) vs. protein concentration.

### 2.3. Statistical Analysis

Statistica 10 PL software was used for statistical analysis. The following characteristics were used for descriptive analysis, characterizing the group of patients: median and IQR values. The distribution of the patients’ data was drawn up and did not meet the criteria for using parametric tests because of its heterogeneity. Therefore, non-parametric tests were used. The Mann–Whitney U test was used to compare two groups of patients. For the comparison of three groups, Dunn’s post-hoc test was used. Due to the lack of normal distribution and group heterogeneity, Spearman’s rank correlation coefficient was used. A significance of differences between percentage structures was calculated using the chi square test. In order to determine the usefulness of the studied proteins as diagnostic markers, the Receiver Operating Characteristic (ROC) curve was plotted, and the area under the curve was calculated to characterize the sensitivity and specificity of the studied parameters. The point closest to (0.1) the approach was used to calculate the ROC optimal cut-off. Univariate and multivariate analyses were performed simultaneously using the Cox regression model. The parameters of the multivariate Cox analysis included age, FIGO staging, grading, EOC subtype and concentrations of those proteins tested that came out statistically significant as risk factors in the univariate analysis, i.e., for serum, they were CD27, CD70 and CD80 concentrations, and for peritoneal fluid, they were BTLA and CD27 concentrations. Analysis of the proteins studied was performed for both serum and peritoneal fluid concentrations. A value of *p* < 0.05 was considered as the statistical significance indicator.

## 3. Results

### 3.1. Serum and Peritoneal Effusion Fluid Concentration of Studied Parameters

In study group A, 31 patients had a diagnosis of serous ovarian cancer, while the remaining 6 patients with non-serous carcinomas were predominantly diagnosed with endometrioid, mucinous and clear cell carcinomas. Of the proteins assayed, only CD27 and CD28 showed statistically significant differences in median serum levels in patients with serous vs. non-serous carcinomas (*p* = 0.03, *p* = 0.04, respectively). However, in peritoneal fluid, we found these differences only for CD27 protein (*p* = 0.04).

The median concentrations of the studied proteins (BTLA, CD27, CD28 and CD80) were statistically significantly higher in serum of patients in the ovarian cancer group (group A) compared to serum concentrations in patients with benign ovarian lesions (group B). It should be mentioned, however, that despite borderline statistical significance (*p* = 0.05), serum CD70 levels were also higher in group A. Peritoneal fluid showed statistically significantly higher median for CD27 and CD70 protein concentrations in the study group compared to the control group (*p* = 0.04, *p* = 0.02, respectively). Table 3 details the concentrations of the studied proteins in serum and peritoneal fluid in group A and group B.

The proteins studied were evaluated for differences in their serum and peritoneal fluid concentrations and selected prognostic factors. Statistically significant higher median serum levels of CD27 and CD80 proteins were found in highly advanced ovarian cancer (stage III–IV) compared to low advanced ovarian cancer (stage I–II) (*p* = 0.02 and *p* = 0.03, respectively). In peritoneal fluid, statistically significantly higher levels were found in stage III–IV patients compared to stage I–II only for CD80 protein (*p* = 0.01) (Table 4).

Significantly higher median serum levels of BTLA, CD27 and CD80 proteins were found in the group of high-grade carcinomas compared to the group of low-grade carcinomas (*p* = 0.01, *p* = 0.01, *p* = 0.03, respectively). In the peritoneal fluid, significantly higher levels in the high-grade group compared to the low-grade group were found for BTLA, CD27, CD70 and CD80 (*p* = 0.04, *p* = 0.03, *p* = 0.01, *p* = 0.04, respectively) (Table 5).

The presence or absence of tumor cells in the peritoneal fluid did not significantly affect the differences in serum concentrations of the proteins studied. In contrast, significant differences were found in the concentrations of proteins in the peritoneal fluid. BTLA and CD70 concentrations were found to be higher in cancers with tumor cells present in the peritoneal fluid (*p* = 0.04, *p* = 0.02, respectively) (Table 6). Similarly, there were no significant differences in serum protein concentrations according to the presence or absence of peritoneal carcinomatosis, and significantly higher concentrations of CD70 and CD80 were shown in peritoneal fluid for peritoneal carcinomatosis (*p* = 0.03, *p* = 0.02) (Table 7).

### 3.2. Correlations between Studied Parameters

Spearman’s rank correlation was used to calculate correlations between concentrations of the proteins studied. A high positive correlation was found between serum concentrations of BTLA and CD27 (r = 0.711, *p* = 0.021), CD27 and CD28 (r = 0.645, *p* = 0.035) and CD27 and CD70 (r = 0.639, *p* = 0.174). A low positive correlation was found between serum concentrations of CD70 and CD28 (r = 0.312, *p* = 0.269). All correlations between serum protein concentrations are included in Table 8.

In peritoneal fluid, high positive correlation was found between CD27 and CD70 (r = 0.694, *p* = 0.056), CD28 and CD80 (r = 0.662, *p* = 0.049) and BTLA and CD27 (r = 0.606, *p* = 0.576). A low positive correlation was found between BTLA and CD80 (r = 0.348, *p* = 0.325) and CD27 and CD80 (r = 0.288, *p* = 0.281). All correlations between peritoneal fluid protein concentrations are included in Table 9.

Spearman’s rank correlation was used to calculate correlations between serum concentrations of the proteins studied and age, menopausal status and BMI. No high positive correlation was found. There was a low positive correlation between age and CD70 (r = 0.175, *p* = 0.034), between BMI and CD27 (r = 0.301, *p* = 0.041) and a moderate positive correlation between menopausal status and CD27 (r = 0.401, *p* = 0.048) and between BMI and CD28 (r = 0.423, *p* = 0.026) (Table 10).

### 3.3. Receiver Operating Characteristic (ROC) Curve for Using BTLA, CD27, CD70, CD28 and CD80 Distinguishing between Ovarian Cancer and Benign Ovarian Lesion

**Figure 1:**
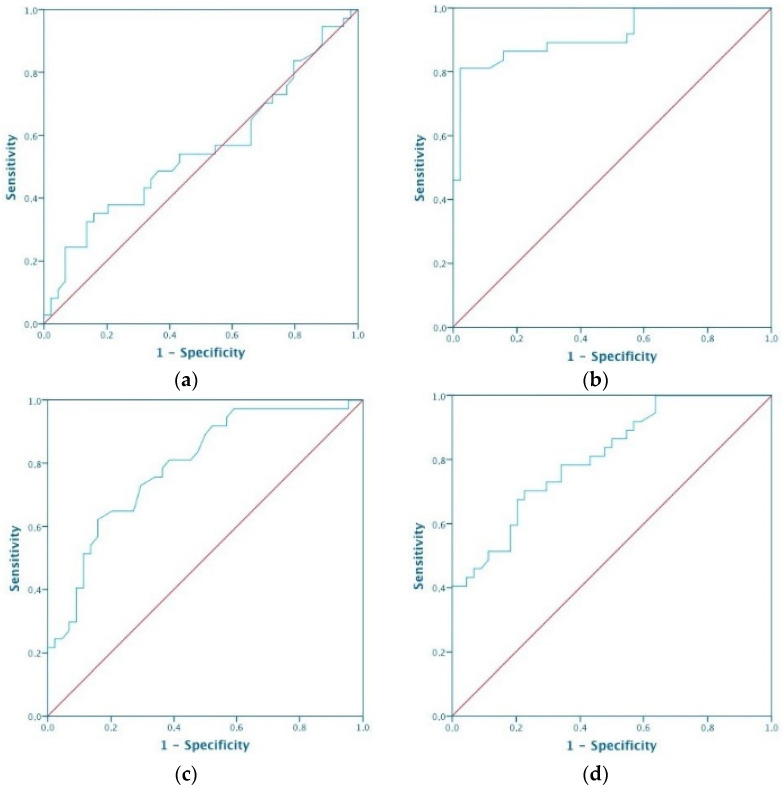

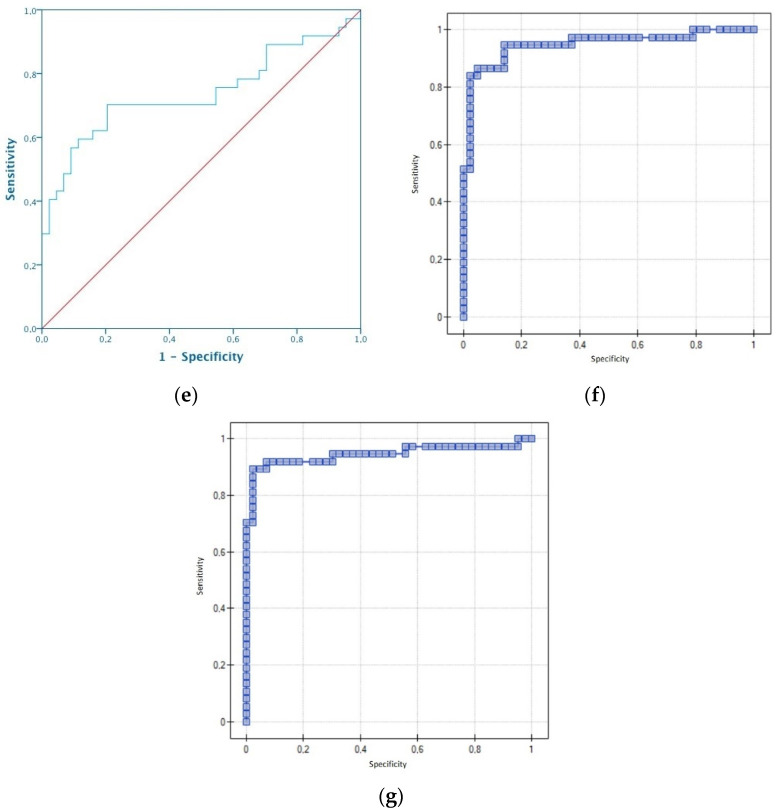
Receiver operating characteristic curve for using (**a**) BTLA, (**b**) CD27, (**c**) CD70, (**d**) CD28, (**e**) CD80, (**f**) CA125 and (**g**) HE4 in distinguishing between ovarian cancer and benign ovarian lesions.

The cutoff values for BTLA, CD27, CD70, CD28 and CD80 that were elevated in the serum of patients with ovarian cancer were calculated using ROC curve analysis. The analysis revealed that when the serum BTLA concentration was 613.3 pg/mL or higher, the sensitivity and specificity were 57 and 72%, respectively (*p* = 0.004). When the serum CD27 concentration was 120.6 pg/mL or higher, the sensitivity and specificity were 66 and 84%, respectively (*p* = 0.014). When the serum CD70 concentration was 4.5 pg/mL or higher, the sensitivity was 79% and the specificity was 51%, but this result was not statistically significant (*p* = 0.057). When the serum CD28 concentration was 113.5 pg/mL, the sensitivity and specificity were 44 and 77%, respectively (*p* = 0.028); and when the serum CD80 concentration was 3.9 pg/mL or higher, the sensitivity and specificity were 63 and 65%, respectively (*p* = 0.041). To compare the diagnostic value of the proteins tested, ROC was also performed for markers used in clinical practice—CA125 and HE4. When the serum CA125 concentration was 49.6 pg/mL or higher, the sensitivity was 95% and the specificity was 81% (*p* = 0.01). When the serum HE4 concentration was 81.2 pg/mL or higher, the sensitivity and specificity were 93 and 87%, respectively (*p* = 0.001) (Table 11). The ROC curves for BTLA, CD27, CD70, CD28, CD80, CA125 and HE4 are shown in Figure 1.

### 3.4. Survival Analysis Using COX Regression

In univariate analysis, the length of PFS (progression-free survival) was affected by staging, primary debulking surgery and preoperative serum levels of CD27 (*p* = 0.021, *p* = 0.006, *p* = 0.048, respectively). It should be noted that only in primary debulking surgery was the HR < 1 (0.92). OS (overall survival) was affected by more factors, including age (*p* = 0.042), staging (*p* = 0.002), grading (*p* = 0.046), EOC subtype (*p* = 0.039) and primary debulking surgery (*p* = 0.048). Preoperative serum levels of CD27 (*p* = 0.021), CD70 (*p* = 0.013) and CD80 (*p* = 0.044) also had an impact on OS length. In multivariate analysis, independent risk factors affecting OS were staging (*p* = 0.022) and CD27 levels (*p* = 0.014). In contrast, independent factors affecting PFS were also staging (*p* = 0.016) and CD27 concentration (*p* = 0.047). A detailed presentation of the impact of the risk factors studied and the serum proteins studied is shown in Table 12.

When analyzing the effect of peritoneal fluid concentrations of the studied proteins through univariate analysis, there was no significant effect of any of the studied proteins on PFS. However, there was an effect of age on PFS (*p* = 0.034). The preoperative effect of peritoneal fluid concentration of the studied proteins for overall survival was different. HR increased for BTLA (*p* = 0.012) and CD27 (*p* = 0.046) affecting OS. In addition, HR was higher at 1.22 for patients with more advanced clinical malignancy (FIGO staging III and IV) (*p* = 0.031). OS was also affected by EOC subtype (*p* = 0.045), where the HR was 1.05 and primary debulking surgery was (*p* = 0.049), but in this case, the HR was 0.94. Multivariate analysis demonstrated that independent risk factors affecting OS were staging (*p* = 0.041) and BTLA levels (*p* = 0.033). In contrast, staging (*p* = 0.021) is also an independent risk factor affecting PFS. A detailed presentation of the effect of the risk factors studied and the proteins studied in the peritoneal fluid is shown in Table 13.

## 4. Discussion

In this study, we focused on the determination of selected proteins in the serum and peritoneal fluid of patients with ovarian cancer. There are little data on the use of BTLA as a diagnostic and prognostic biomarker in ovarian cancer, especially when it comes to measuring protein levels in peritoneal fluid. Previous studies have focused on measuring BTLA in tumor tissue, indicating that it may predict poor outcome for EOC patients [7]. BTLA expression on CD4+ T cells and CD8+ T cells in the tumor microenvironment of EOC was also studied, which was 37.6% and 15.7%, respectively [12]. Although these data provide important information about the biomolecular characteristics of ovarian cancer, we would like to emphasize the relevance of our study in evaluating BTLA as a diagnostic and prognostic factor. We observed significantly higher serum BTLA levels in patients with ovarian cancer (1418.8 pg/mL vs. 454.7 pg/mL in patients with benign ovarian lesions). Furthermore, in our analyses, we showed that BTLA (levels above the cut-off in peritoneal fluid) is a prognostically unfavorable factor and is associated with shorter survival. CD27 has been used for biomolecular characterization of ovarian cancer in previous studies [13,14]. As shown by Nielsen et al., in high-grade serous ovarian tumors, CD20(+) tumor-infiltrating lymphocytes (TIL) have an antigen-experienced but atypical CD27(−) memory B cell phenotype [13]. The authors indicate that the association between CD20(+), TIL and patient survival may reflect a supportive role in cytolytic immune responses [13]. The research on CD27 seems to be even more important because in the context of an effective cancer vaccination, CD27 agonism with anti-PD-1 therapy further improves treatment efficacy [15]. Our study demonstrated significantly higher levels of CD27 in patients with ovarian cancer, both in serum and peritoneal fluid, which, in comparison with previous studies, shows a new clinical aspect of this marker. Moreover, as a diagnostic biomarker, it has 84% specificity (0.014 95% CI, *p* = 0.014). CD27 also affects progression-free survival and overall survival. For CD70, which forms a molecular pathway with CD27, we demonstrated this increased concentration in the peritoneal fluid of ovarian cancer patients. Additionally, we found high positive correlation between CD27 and CD70, but this result was not statistically significant. Nevertheless, this correlation may be due to common axis. As studies show, elevated expression of CD70 is associated with drug resistance and poor prognosis, but in this case, the expression was studied in advanced ovarian cancer specimens [16]. Another study also identifies CD70 molecule as a marker associated with platinum resistance [17]. In our study, according to univariate analysis, increased serum CD70 levels were associated with poor prognosis in terms of OS. The next molecules we examined were those of the CD28–CD80 axis. Although we did not find a strong correlation between the studied proteins, we found higher serum levels of these proteins in patients with ovarian cancer compared to controls. The analyses showed no significant effect of these molecules on progression-free survival and overall survival. However, attention should be paid to these molecules because of their potential targets in anticancer therapy, involving a class of CD28-costimulatory bispecific antibodies with the emerging class of TSAxCD3 bispecifics [18]. CD28 have also been characterized as molecules that co-upregulated CXCR3 and CXCR4 and enhanced their migration toward universally expressed chemokines [19]. In turn, a study by Conejo-Garcia et al. examined ovarian expression of NKG2D Ligand Letal as a factor promoting the survival and expansion of CD28 antitumor T cells [20]. Furthermore, CD28 expression has also been studied in ascites tumor-infiltrating lymphocytes [21,22]. On the other hand, 2021 findings encourage incorporating CD28 signaling into chimeric antigen receptor (CAR) design for adoptive T cell treatment of solid tumors [23]. Although the studies we have presented here focus on presenting the proteins studied in the context of use in ovarian cancer diagnosis and prognosis, there is still a need for more research in this area. Previous studies have investigated CD80 from different aspects and with different uses, but there are few data presenting CD80 as a molecule used in the diagnosis and prognosis of ovarian cancer, where the test material is patient serum and peritoneal fluid [24,25,26,27]. For example, CD80 expression was studied, and high levels of CD80 expression were observed on Gr-1+ CD11b+ ovarian cancer cells [28]. It was concluded that CD80-dependent responses to myeloid suppressor cells may contribute to tumor tolerance and the progression of ovarian carcinoma [28]. The few data in the literature on the use of the proteins we studied as diagnostic and prognostic biomarkers suggest the uniqueness of our study. Nevertheless, our findings need to be confirmed in other studies. On the basis of this study, we believe that the proteins we evaluated play an important role as biomarkers in ovarian cancer. We also tested the diagnostic utility of the proteins in daily clinical practice—CA125 and HE4. In our study group, we showed that both proteins have better diagnostic value, taking into account sensitivity and specificity, compared to the tested immune response proteins. Unfortunately, the drawback of our study is the lack of validation, and our results are based on statistical analysis. Therefore, we have plans to continue our study to verify whether the proteins we tested will prove successful in clinical practice. The small group size and the possibility of residual confounding may also have influenced the results of this study, so our results need all the more to be confirmed in other studies with larger study groups. Nevertheless, we believe that the proteins we studied have diagnostic potential and may, in the future, support the diagnostic management of ovarian cancer.

In summary, as this study demonstrates, CD27 can be used as a biomarker to distinguish ovarian cancer from benign ovarian lesions, as it has a specificity of 84% when the cut-off is 120.6 pg/mL. BTLA, CD28 and CD80 may also contribute to the diagnosis of ovarian cancer, but their possible insufficient sensitivity and specificity as diagnostic markers should be taken into account. Hence, our findings need to be confirmed in other studies. On the basis of multivariate analysis, we conclude that CD27 determined in serum is an unfavorable prognostic factor, affecting PFS and OS. Based on the multivariate analysis performed for peritoneal fluid protein concentrations, we conclude that BTLA is an unfavorable prognostic factor, further affecting OS.

## 5. Conclusions

CD27 should be considered as a potential biomarker in the diagnosis of ovarian cancer. CD27, determined in serum, is an unfavorable prognostic factor for ovarian cancer. BTLA, determined in peritoneal fluid, is an unfavorable prognostic factor for ovarian cancer.

## Figures and Tables

**Table 1:** Clinicopathological characteristics for the enrolled ovarian cancer patients.

Characteristic	Value	Number of Patients
EOC subtype	Serous	31
	Endometrioid	4
	Mucinous	1
	Clear cell	1
FIGO staging	I–II	7
	III–IV	30
Grade	Low	13
	High	24
Neoplastic cells in peritoneal fluid	Yes	27
	No	10
Peritoneal carcinomatosis	Yes	26
	No	11
Primary debulking surgery	Total	14
	Subtotal	12
Interval debulking surgery	Total	8
	Subtotal	3
Chemotherapy	Neoadjuvant	11
	Adjuvant	26

**Table 2:** Clinicodemographic characteristics of the patients studied.

Clinicodemographic Characteristics	Total Cohort (*n* = 79)	Cases (*n* = 37)	Controls (*n* = 42)	*p*-Value
Median (IQR)				
Age (years old)	54.7 (50–58)	60.2 (55–64)	47.6 (43–53)	0.046
BMI (kg/m^2^)	26.9 (21.8–29.4)	27.8 (23.1–31.6)	25.2 (22.7–28.6)	0.081
Number (%)				
Age (years old)				0.099
<65	51 (64.6%)	16 (43.2%)	25 (59.5%)	
≥65	28 (35.4%)	21 (56.8%)	17 (40.5%)	
BMI				0.002
<25	54 (68.4%)	19 (51.4%)	35 (83.3%)	
≥25	25 (21.6%)	18 (48.6%)	7 (16.7%)	
Menopausal status				0.005
Premenopausal	31 (39.2%)	11 (29.7%)	20 (47.6%)	
Postmenopausal	48 (60.8%)	26 (71.3%)	22 (52.4%)	

**Table 3:** Levels of serum and peritoneal fluid concentrations of the studied proteins in ovarian cancer group and benign ovarian lesion group.

Characteristics		BTLA Serum/ BTLA Effusion (pg/mL)	CD27 Serum/ CD27 Effusion (pg/mL)	CD70 Serum/ CD70 Effusion (pg/mL)	CD28 Serum/ CD28 Effusion (pg/mL)	CD80 Serum/ CD80 Effusion (pg/mL)
Ovarian cancer group	median	1418.8/815.7	136.8/138.8	5.8/7.9	121.1/121.6	5.5/5.2
	IQR	621.2/324.5	27.6/31.1	0.7/1.1	19.3/26.3	0.8/0.9
Benign ovarian lesion group	median	454.7/767.1	112.8/114.5	3.9/5.4	110.7/118.2	2.6/4.2
	IQR	108.3/196.3	22.0/24.1	0.3/1.1	19.3/20.9	0.5/0.9
*p* value		0.01/0.418	0.03/0.04	0.05/0.02	0.02/0.1	0.01/0.09

**Table 4:** Levels of serum and peritoneal fluid concentrations of the studied proteins in the compared tumor clinical stage groups.

Characteristics		BTLA Serum/ BTLA Effusion (pg/mL)	CD27 Serum/ CD27 Effusion (pg/mL)	CD70 Serum/ CD70 Effusion (pg/mL)	CD28 Serum/ CD28 Effusion (pg/mL)	CD80 Serum/ CD80 Effusion (pg/mL)
Stage I–II	median	1096.8/1034.5	112.2/106.7	5.0/6.3	132.7/130.9	4.9/5.0
	IQR	312.1/224.8	17.4/13.9	0.7/1.2	12.2/22.4	0.7/1.0
Stage III–IV	median	1202.4/1108.4	142.1/125.5	5.5/6.9	123.4/117.3	5.3/7.2
	IQR	401.6/245.2	25.1/20.7	0.8/1.3	18.6/17.9	0.5/0.9
*p* value		0.61/0.08	0.02/0.05	0.06/0.07	0.2/0.05	0.03/0.01

**Table 5:** Levels of serum and peritoneal fluid concentrations of the studied proteins in the compared grading groups.

Characteristics		BTLA Serum/ BTLA Effusion (pg/mL)	CD27 Serum/ CD27 Effusion (pg/mL)	CD70 Serum/ CD70 Effusion (pg/mL)	CD28 Serum/ CD28 Effusion (pg/mL)	CD80 Serum/ CD80 Effusion (pg/mL)
Low-Grade	median	1126.2/1111.8	119.6/118.1	5.8/6.1	123.1/124.9	5.1/5.4
	IQR	347.2/318.9	16.6/21.2	0.8/1.1	30.7/25.6	0.9/1.2
High-Grade	median	1318.2/1344.4	140.4/140.1	6.0/6.9	126.7/130.3	7.0/7.1
	IQR	232.5/302.2	22.8/24.6	0.4/0.9	16.8/22.5	0.8/0.6
*p* value		0.01/0.04	0.01/0.03	0.06/0.01	0.08/0.4	0.03/0.04

**Table 6:** Levels of serum and peritoneal fluid concentrations of studied proteins in relation to the presence of neoplastic cells in the peritoneal fluid.

Characteristics		BTLA Serum/ BTLA Effusion (pg/mL)	CD27 Serum/ CD27 Effusion (pg/mL)	CD70 Serum/ CD70 Effusion (pg/mL)	CD28 Serum/ CD28 Effusion (pg/mL)	CD80 Serum/ CD80 Effusion (pg/mL)
Neoplastic cells in peritoneal fluid	median	1267.2/1371.3	128.1/122.6	5.9/6.3	121.3/133.3	5.8/4.3
	IQR	235.1/327.7	34.2/22.6	1.2/0.9	29.6/31.7	1.2/0.5
No neoplastic cells in peritoneal fluid	median	1202.4/1108.7	125.5/121.9	5.5/5.0	120.4/124.8	6.0/5.3
	IQR	268.5/221.7	19.9/23.7	0.6/0.7	40.6/31.4	1.4/1.1
*p* value		0.23/0.04	0.18/0.32	0.14/0.02	0.35/0.29	0.61/0.53

**Table 7:** Levels of serum and peritoneal fluid concentrations of studied proteins in relation to the presence of peritoneal carcinomatosis.

Characteristics		BTLA Serum/ BTLA Effusion (pg/mL)	CD27 Serum/ CD27 Effusion (pg/mL)	CD70 Serum/ CD70 Effusion (pg/mL)	CD28 Serum/ CD28 Effusion (pg/mL)	CD80 Serum/ CD80 Effusion (pg/mL)
Peritoneal carcinomatosis	median	1321.5/1324.6	130.2/129.1	6.8/7.2	118.2/125.7	5.8/7.9
	IQR	203.5/241.6	20.8/25.1	0.7/0.9	21.8/22.5	0.6/1.0
No peritoneal carcinomatosis	median	1250.1/1286.7	126.6/122.2	6.0/5.4	126.1/118.7	5.2/4.8
	IQR	368.9/333.2	30.6/33.8	1.2/1.3	31.6/29.7	1.5/1.2
*p* value		0.27/0.44	0.24/0.31	0.05/0.03	0.42/0.18	0.33/0.02

**Table 8:** The correlations between serum concentrations of BTLA, CD27, CD70, CD28 and CD80, presented as Spearman ranges‚ r correlation coefficient/*p*-value.

Variable	BTLA	CD27	CD70	CD28
BTLA	-	-	-	-
CD27	0.711/0.021	-	-	-
CD70	0.515/0.082	0.639/0.174	-	-
CD28	0.589/0.201	0.645/0.035	0.312/0.269	-
CD80	0.511/0.47	0.397/0.165	0.595/0.38	0.499/0.322

**Table 9:** The correlations between BTLA, CD27, CD70, CD28 and CD80 concentrations in peritoneal fluid, presented as Spearman ranges‚ r correlation coefficient/*p*-value.

Variable	BTLA	CD27	CD70	CD28
BTLA	-	-	-	-
CD27	0.606/0.576	-	-	-
CD70	0.421/0.097	0.694/0.056	-	-
CD28	0.519/0.251	0.586/0.411	0.407/0.196	-
CD80	0.348/0.325	0.288/0.281	0.526/0.380	0.662/0.049

**Table 10:** The correlations between serum concentrations of BTLA, CD27, CD70, CD28 and CD80 and age, menopausal status and BMI, presented as Spearman ranges‚ r correlation coefficient, *p*-value.

Variable		BTLA	CD27	CD70	CD28	CD80
Age (mean)	r correlation coefficient	0.321	0.489	0.175	0.298	0.521
	*p*-value	0.089	0.126	0.034	0.301	0.051
Menopausal status	r correlation coefficient	0.367	0.401	0.196	0.387	0.267
	*p*-value	0.091	0.048	0.067	0.324	0.181
BMI	r correlation coefficient	0.237	0.301	0.199	0.423	0.314
	*p*-value	0.187	0.041	0.094	0.026	0.465

**Table 11:** The diagnostic values of BTLA, CD27, CD70, CD28, CD80, CA125 and HE4 for patients with ovarian cancer.

Marker	AUC (95% CI)	Sensitivity (%)	Specificity (%)	*p*-Value	Cut-Off Value (pg/mL)
CA125	0.95	95	81	0.01	49.6
HE4	0.94	93	87	0.001	81.2
CD27	0.915	66	84	0.014	120.6
CD70	0.821	79	51	0.057	4.5
CD28	0.808	44	77	0.028	113.5
CD80	0.727	63	65	0.041	3.9
BTLA	0.553	57	72	0.004	613.3

**Table 12:** Univariate and multivariate Cox regression model for serum concentrations of the proteins studied.

Univariate Analysis						
Variable	PFS			OS		
	HR	95% CI	*p*-Value	HR	95% CI	*p*-Value
Age (above vs. below median)	1.32	1.09–1.36	0.052	1.06	1.02–1.13	0.042
FIGO staging (III and IV vs. I and II)	1.47	1.28–1.50	0.021	1.37	1.28–1.39	0.002
Grade (high vs. low)	1.21	1.16–1.28	0.433	1.26	1.24–1.30	0.046
EOC subtype (serous vs. non-serous)	1.08	0.98–1.19	0.086	1.11	1.02–1.15	0.039
Primary debulking surgery (total vs. subtotal)	0.92	0.89–0.95	0.006	0.90	0.87–0.91	0.048
Interval debulking surgery (total vs. subtotal)	0.98	0.96–1.08	0.236	0.99	0.98–1.06	0.059
BTLA level (above vs. below cut-off)	1.21	1.13–1.26	0.069	1.29	1.22–1.37	0.071
CD27 (above vs. below cut-off)	1.28	1.25–1.30	0.048	1.33	1.30–1.41	0.021
CD70 (above vs. below cut-off)	1.23	1.17–1.29	0.051	1.30	1.27–1.34	0.013
CD28 (above vs. below cut-off)	1.06	1.01–1.15	0.132	1.14	1.09–1.19	0.086
CD80 (above vs. below cut-off)	1.40	1.33–1.41	0.242	1.18	1.14–1.20	0.044
**Multivariate Analysis**						
	**PFS**			**OS**		
	**HR**	**95% CI**	***p*-Value**	**HR**	**95% CI**	***p*-Value**
Age (above vs. below median)	1.15	1.07–1.20	0.091	1.23	1.17–1.28	0.054
FIGO staging (III and IV vs. I and II)	1.21	1.18–1.24	0.016	1.36	1.26–1.38	0.022
Grade (high vs. low)	1.27	1.26–1.38	0.211	1.33	1.31–1.35	0.072
EOC subtype (serous vs. non-serous)	1.12	1.11–1.16	0.077	1.18	1.16–1.21	0.153
CD27 (above vs. below cut-off)	1.26	1.21–1.29	0.047	1.20	1.15–1.22	0.014
CD70 (above vs. below cut-off)	1.09	1.05–1.12	0.211	1.13	1.10–1.19	0.196
CD80 (above vs. below cut-off)	1.14	1.10–1.19	0.108	1.12	1.05–1.13	0.088

**Table 13:** Univariate and multivariate Cox regression model for concentrations of studied proteins in peritoneal fluid.

Univariate Analysis						
Variable	PFS			OS		
	HR	95% CI	*p*-Value	HR	95% CI	*p*-Value
Age (above vs. below median)	1.25	1.22–1.32	0.034	1.17	1.09–1.19	0.058
FIGO staging (III and IV vs. I and II)	1.32	1.29–1.38	0.051	1.22	1.14–1.32	0.031
Grade (high vs. low)	1.15	1.10–1.24	0.106	1.09	1.08–1.12	0.066
EOC subtype (serous vs. non-serous)	1.03	0.97–1.05	0.348	1.05	1.01–1.11	0.045
Primary debulking surgery (total vs. subtotal)	0.97	0.95–1.07	0.122	0.94	0.93–1.01	0.049
Interval debulking surgery (total vs. subtotal)	0.86	0.80–0.89	0.673	0.93	0.89–0.94	0.177
BTLA (above vs. below cut-off)	1.20	1.14–1.28	0.061	1.14	1.07–1.20	0.012
CD27 (above vs. below cut-off)	1.09	1.06–1.13	0.248	1.21	1.13–1.22	0.046
CD70 (above vs. below cut-off)	1.08	1.06–1.15	0.111	1.11	1.07–1.20	0.145
CD28 (above vs. below cut-off)	0.96	0.94–1.07	0.232	1.02	0.99–1.08	0.098
CD80 (above vs. below cut-off)	1.11	1.06–1.12	0.149	1.19	1.15–1.22	0.155
**Multivariate Analysis**						
	**PFS**			**OS**		
	**HR**	**95% CI**	***p*-Value**	**HR**	**95% CI**	***p*-Value**
Age (above vs. below median)	1.16	1.13–1.20	0.076	1.21	1.18–1.24	0.152
FIGO staging (III and IV vs. I and II)	1.23	1.18–1.26	0.021	1.29	1.27–1.33	0.041
Grade (high vs. low)	1.25	1.21–1.27	0.142	1.22	1.19–1.27	0.093
EOC subtype (serous vs. non-serous)	1.09	1.06–1.12	0.233	1.05	1.01–1.13	0.126
BTLA (above vs. below cut-off)	1.20	1.15–1.21	0.161	1.26	1.25–1.31	0.033
CD27 (above vs. below cut-off)	1.07	1.04–1.09	0.142	1.11	1.08–1.16	0.132

## Data Availability

The data presented in this study are available on request from corresponding author, M.K., upon reasonable request.
